# Textbook outcome for esophageal cancer surgery: an international consensus-based update of a quality measure

**DOI:** 10.1093/dote/doab011

**Published:** 2021-03-22

**Authors:** Marianne C Kalff, Mark I van Berge Henegouwen, Suzanne S Gisbertz

**Affiliations:** Department of Surgery, Amsterdam UMC, Location AMC, Cancer Center Amsterdam, Amsterdam, The Netherlands; Department of Surgery, Amsterdam UMC, Location AMC, Cancer Center Amsterdam, Amsterdam, The Netherlands; Department of Surgery, Amsterdam UMC, Location AMC, Cancer Center Amsterdam, Amsterdam, The Netherlands

**Keywords:** esophageal cancer, esophagectomy, textbook outcome

## Abstract

Textbook outcome for esophageal cancer surgery is a composite quality measure including 10 short-term surgical outcomes reflecting an uneventful perioperative course. Achieved textbook outcome is associated with improved long-term survival. This study aimed to update the original textbook outcome based on international consensus. Forty-five international expert esophageal cancer surgeons received a personal invitation to evaluate the 10 items in the original textbook outcome for esophageal cancer surgery and to rate 18 additional items divided over seven subcategories for their importance in the updated textbook outcome. Items were included in the updated textbook outcome if ≥80% of the respondents agreed on inclusion. In case multiple items within one subcategory reached ≥80% agreement, only the most inclusive item with the highest agreement rate was included. With a response rate of 80%, 36 expert esophageal cancer surgeons, from 34 hospitals, 16 countries, and 4 continents responded to this international survey. Based on the inclusion criteria, the updated quality indicator ‘textbook outcome for esophageal cancer surgery’ should consist of: tumor-negative resection margins, ≥20 lymph nodes retrieved and examined, no intraoperative complication, no complications Clavien–Dindo ≥III, no ICU/MCU readmission, no readmission related to the surgical procedure, no anastomotic leakage, no hospital stay ≥14 days, and no in-hospital mortality. This study resulted in an international consensus-based update of a quality measure, textbook outcome for esophageal cancer surgery. This updated textbook outcome should be implemented in quality assurance programs for centers performing esophageal cancer surgery, and could standardize quality measures used internationally.

## INTRODUCTION

Surgical esophageal cancer treatment is associated with considerable postoperative morbidity and mortality.^1^^,^^2^ In order to improve quality of care, and subsequently decrease postoperative morbidity and mortality, quality assurance measures may be crucial.^3–5^ Textbook outcome for gastro-esophageal cancer surgery was defined in 2017 and includes 10 short-term surgical outcomes selected by expert opinion within the scientific committee of the obligatory nationwide Dutch Upper GI Cancer Audit (DUCA; Table 1).^4^ It was introduced as a tool for quality assurance of gastro-esophageal cancer surgery, reflecting the quality of surgical care from surgery until the short-term postoperative phase. The DUCA provides periodic benchmarked feedback regarding hospital performance, including textbook outcome rate, to all centers performing gastro-esophageal cancer surgery in the Netherlands, stimulating surgical quality improvement.

**Table 1:** Original and updated textbook outcome variables for esophageal cancer surgery

Variables in original textbook outcome	Variables in updated textbook outcome
Complete resection according to the surgeon at the end of surgery	
Tumor-negative resection margins (R0)	Tumor-negative resection margins (R0)
≥ 15 lymph nodes retrieved and examined	≥ 20 lymph nodes retrieved and examined
No intraoperative complication	No intraoperative complication
No complication of ≥ CD II	No complication of ≥ CD III
No reintervention ≤30 days after surgery	
	No anastomotic leakage (all ECCG grades)
No ICU/MCU readmission ≤30 days after surgery	No ICU/MCU readmission
No hospital stay ≥21 days	No hospital stay ≥14 days
No in-hospital and no 30-day mortality	No in-hospital mortality
No hospital readmission ≤30 days after discharge	No readmission related to the surgical procedure

CD, Clavien–Dindo; ECCG, Esophagectomy Complications Consensus Group; ICU, intensive care unit; MCU, medium care unit.

Adjacent to its use for quality assurance purposes in the obligatory Dutch audit, textbook outcome for gastro-esophageal cancer surgery is used in multiple research projects,^6–8^ not limited to Dutch origin.^9^^,^^10^ Previous studies showed the correlation of achieved textbook outcome with increased survival,^6^^,^^8^ the increase in textbook outcome rate during the implementation of minimally invasive surgery,^7^ and its relation with case volume.^9^ Although textbook outcome is being increasingly recognized as a valuable quality indicator, controversy concerning some of the items used in this composite measure exists; some items show overlapping definitions and others may need to be redefined. Within the original textbook outcome, severe postoperative morbidity is defined as Clavien–Dindo grade II or higher, however, as this is by definition treated noninvasively, this may not be the best cutoff for severe morbidity.^11^ Furthermore, the inclusion of specific post-esophagectomy morbidity, such as anastomotic leakage or anastomotic stricture, could increase the value of this quality indicator specifically for esophageal cancer surgery. Moreover, although other composite quality measures are used for esophageal cancer surgery internationally, none are based on international consensus.^12^^,^^13^

The aim of this survey was to consult international experts on their opinion of what constitutes a textbook outcome for esophageal cancer surgery and to subsequently update the original textbook outcome to an international consensus-based composite quality measure, which could facilitate and stimulate surgical quality improvement based on (international) benchmarking.

## METHODS

### Participants

In order to update the original definition of textbook outcome for esophageal cancer surgery based on international consensus, a survey was conducted inquiring international experts on their opinion of what constitutes a textbook outcome for esophageal cancer surgery. Forty-five renowned international expert esophageal cancer surgeons were selected from centers participating in the TIGER study, an ongoing international observational cohort study on esophageal cancer and the distribution of lymph node metastases.^14^ In July 2019, these surgeons received a personal invitation to participate in the online survey, with reminders after 2 and 4 weeks for nonresponders.

### Survey

Surgeons were asked to re-evaluate the items included in the original textbook outcome and to rate additional items for their importance in the updated textbook outcome. Additionally, general information on respondents’ age, years of experience as gastro-esophageal surgeon, hospital volume and personal volume were asked. Potentially new textbook outcome items were included based on a Pubmed search of the current literature on short-term outcomes of esophageal cancer surgery (conducted in June 2019). The search contained the following terms and their synonyms: esophageal cancer, esophagectomy, morbidity, and mortality. Potential textbook outcome items identified included lymph node harvest, surgical resection margins, severity of postoperative morbidity, anastomotic leakage, cardiac and pulmonary complications, length of hospital stay, and postoperative mortality. In total, 28 potential textbook outcome items were included in the survey, divided over five categories; oncological quality, general surgical complications, complications related to esophageal cancer surgery, length of hospital stay, and mortality (Table 2). When items included in the survey showed much resemblance, they were combined into one subcategory, resulting in seven subcategories; lymph node harvest, severe postoperative complications, ICU/MCU admission, hospital readmission, anastomotic leakage, length of hospital stay, and postoperative mortality. From these subcategories, only one item per subcategory could be included in the updated textbook outcome. Every category was followed by a comment field providing responding surgeons the opportunity to comment on the included items of that category, and to propose other items to be included.

**Table 2:** International survey items and respondents’ agreement percentages per item and subcategory

Survey categories, subcategories, and items			Respondents’ agreement rate	
			Round 1	Round 2
Oncological quality		Radical resection according to the surgeon at the end of surgery^†^	72% (26/36)	
		Tumor-negative resection margins^†^	97% (35/36)	
	*Lymph node harvest*		*94% (34/36)*	
		At least 15 lymph nodes retrieved and examined^†^	56% (20/36)	28% (9/32)
		At least 20 lymph nodes retrieved and examined	61% (22/36)	38% (12/32)
		At least 25 lymph nodes retrieved and examined	56% (20/36)	34% (11/32)
General surgical complications		No intraoperative complication^†^	92% (33/36)	
	*Severe postoperative complication (general)*			*97% (35/36)*	
		No complication of CD II or higher^†^	44% (16/36)	
		No complication of CD III or higher	83% (30/36)	
		No complication of CD IV or V	44% (16/36)	
		No re-intervention (surgical / endoscopic / radiologic)^†^	83% (30/36)	
	*ICU/MCU admission*		*81% (29/36)*	
		No ICU/MCU readmission^†^	72% (28/36)	66% (21/32)
		No ICU/MCU stay longer than 1 day	25% (9/36)	6% (2/32)
		No ICU/MCU stay longer than 1 day and no ICU/MCU readmission	44% (16/36)	28% (9/32)
	*Hospital readmission*		*97% (35/36)*	
		No readmission within 30 days after discharge^†^	81% (29/36)	
		No readmission related to the surgical procedure	83% (30/36)	
Post-esophagectomy complications		Pneumonia	59% (25/36)	
		Atrial dysrhythmia	42% (15/36)	
		Anastomotic stricture requiring dilatation <90 days after surgery	61% (22/36)	
	*Anastomotic leakage*			*100% (36/36)*	
		No anastomotic leakage (all grades according to ECCG)	86% (31/36)	
		No anastomotic leakage (grade 2 or higher according to ECCG)	75% (27/36)	
		No anastomotic leakage (grade 3 according to ECCG)	58% (21/36)	
Length of hospital stay	*Length of hospital stay*		*92% (33/36)*	
		No hospital stay longer than 10 days	42% (15/36)	34% (11/32)
		No hospital stay longer than 14 days	58% (21/36)	47% (15/32)
		No hospital stay longer than 21 days^†^	61% (22/36)	19% (6/32)
Mortality	*Postoperative mortality*		*97% (35/36)*	
		No in-hospital mortality	89% (32/36)	
		No 30-day mortality	78% (28/36)	
		No in-hospital and no 30-day mortality^†^	86% (31/36)	
		No 90-day mortality	81% (29/36)	

^†^Items (*n* = 10) in original version of textbook outcome for gastro-esophageal cancer. Text in italics indicate subcategories of resembling items. Values in italics indicate agreement on inclusion of items from the subcategories.

### Statistical analysis

Surgeons where asked to score whether they agreed on the inclusion of each of the items in the updated textbook outcome on a 5-point Likert scale. Items were included in the updated textbook outcome when ≥80% of the surgeons agreed on inclusion of that item. If multiple items regarding the same postoperative outcome, i.e. belonging to the same subcategory, reached ≥80% agreement, only the most inclusive item with the highest agreement rate was included. When experts agreed ≥80% on including an item from a subcategory, but no ≥80% agreement was achieved for one of the items within that subcategory, all responding experts were contacted again to vote for the inclusion of one of the items from that subcategory. Prior to scoring the items within these subcategories, responding experts were informed on the respective agreement rates in the first round of the survey. From the subcategories once more presented, the item per subcategory receiving the most votes was included in the updated textbook outcome. Agreement rates were reported as numbers with corresponding percentages. The survey was designed using Google Forms (Google LLC, Mountain View, Californië, USA), and data were converted to Excel (version 2016; Microsoft Corporation, Redmond, WA, USA).

## RESULTS

**Fig. 1:**
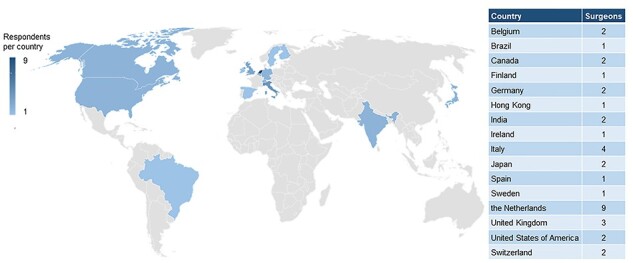
Worldwide distribution of survey respondents.

**Fig. 2:**
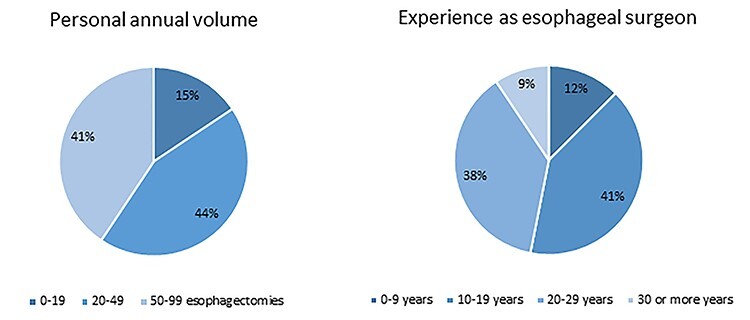
Overview of experience years and personal volume of responding surgeons.

Of the 45 expert esophageal cancer surgeons approached, 36 responded to the survey, resulting in a response rate of 80%. These 36 surgeons came from 34 different hospitals spread over 16 countries and 4 continents (Fig. 1). The majority of surgeons originated from Europe (*n* = 26), followed by Asia (*n* = 5), Northern America (*n* = 4), and Southern America (*n* = 1). Among the responding surgeons, the mean age was 53.7 years (SD 7.0) and the mean experience in gastro-esophageal surgery was 18.7 years (SD 7.8). The mean hospital volume was 79 (SD 44.1) esophagectomies per year. The mean personal annual volume of the responding surgeons was 46 (SD 26.7) esophagectomies per year. Figure 2 shows an overview of the experience years and personal volume of responding surgeons.

Six out of 28 survey items qualified as short-term outcomes required to achieve the updated quality indicator ‘textbook outcome for esophageal cancer surgery’ based on the minimum agreement rate of 80%; tumor-negative resection margins (97%), no intraoperative complication (92%), no complication of Clavien–Dindo III or higher (83%), no readmission related to the surgical procedure (83%), no anastomotic leakage [all grades according to ECCG] (86%),^15^ and no in-hospital mortality (89%).

Most surgeons (81%–94%) agreed on inclusion of items from the ‘lymph node harvest’, ‘ICU/MCU admission’ and ‘length of hospital stay’ subcategories, although no initial agreement was achieved for one of the items within each subcategory. According to the second part of the survey, one item from every of these three subcategories qualified as a short-term outcome required to achieve the updated textbook outcome. About 66% of the surgeons responding in the second part agreed on the inclusion of ‘no ICU/MCU readmission’. Twenty-three of 32 (72%) responding surgeons agreed on a higher minimum lymph node yield required to achieve textbook outcome than present in the original textbook outcome, with at least 20 lymph nodes retrieved and examined as the most frequent preferred cutoff (38%). Twenty-six of 32 (81%) responding surgeons agreed on a shorter maximum hospital stay required to achieve textbook outcome than present in the original textbook outcome, with a maximum hospital stay of 14 days as the most frequent preferred cutoff (47%).

An overview of survey items, and respondents’ agreement percentages per item and subcategory is presented in Table 2.

## DISCUSSION

The use of quality assurance measures may be crucial in improving quality of surgical esophageal cancer treatment. The current study aimed to update the textbook outcome for esophageal cancer surgery to an international consensus-based composite quality measure.

Based on the agreement of 36 international expert esophageal cancer surgeons, the updated textbook outcome for esophageal surgery consists of nine items; tumor-negative resection margins, at least 20 lymph nodes retrieved and examined, no intraoperative complication, no complication Clavien–Dindo grade III or higher, no ICU/MCU readmission, no readmission related to the surgical procedure, no anastomotic leakage [all grades according to ECCG], no hospital stay longer than 14 days, and no in-hospital mortality.

Of the nine items included in the updated textbook outcome, three were already present in the original textbook outcome and five concerned an altered definition based on international consensus. The main changes to the original textbook outcome for esophageal cancer surgery consists of the addition of ‘anastomotic leakage [all grades]’ as a specific post-esophagectomy outcome, and the omission of ‘complete resection as judged by the surgeon’ and ‘no re-intervention’. For the five items altered, the cut-off value was changed based on international expert opinion; the definition of severe postoperative morbidity changed from Clavien–Dindo II or higher to Clavien–Dindo III or higher, the minimum lymph node harvest required to achieve textbook outcome increased from 15 to 20 lymph nodes, the maximum length of hospital stay decreased from 21 to 14 days, hospital readmission changed from within 30 days after surgery to no readmission related to the surgical procedure, and postoperative mortality changed from a combined in-hospital and 30-day mortality to solely no in-hospital mortality.

Whereas other quality indicators also include treatment characteristics, such as the application of neo-adjuvant treatment,^12^ both the original and the updated textbook outcome only include surgical outcomes. The quality of medical care can be assessed by evaluating and comparing structure, process and outcomes of the provided health care, of which Donabedian *et al.* appointed the assessment of health care outcomes as the ultimate quality of care validator.^16^ As such, no treatment specifications were included in this survey. Furthermore, the outcomes included were restricted to short-term outcomes, first because many hospitals and audit registries are limited to the short-term postoperative phase, and second, more important, because long-term outcomes impair the Plan-Do-Check-Act cycle method to convert observed outcomes into practical steps for quality improvement. Importantly, textbook outcome was found to be associated with superior long-term survival in a setup were participating centers received benchmarked information on their performance based on short-term surgical outcomes.^6^ Adjacent to this outcome measure, treatment characteristics representative of a ‘textbook process’, e.g. the administration of neo-adjuvant treatment, use of minimally invasive surgery, and enhanced recovery protocols should still be recorded. Furthermore, variation in textbook outcome rates between hospitals may reflect differences in case-mix variation. For example, more squamous cell carcinomas and higher ASA scores are associated with a lower textbook outcome rate.^8^ These case-mix factors, among others, can also reflect the expertise of a hospital and should be considered when interpreting and comparing textbook outcome rates.

Changes to the original textbook outcome seem to follow some of the important new findings regarding esophageal cancer treatment in literature. The inclusion of anastomotic leakage as a post-esophagectomy complication, could increase the value of textbook outcome as a specific esophageal cancer surgery quality indicator, especially since a recent study found anastomotic leakage to be associated with survival.^17^ The increase in minimum lymph node harvest required to achieve textbook outcome is in line with the increasing evidence that an extensive lymphadenectomy is associated with improved survival and optimized pathological staging, also after neo-adjuvant therapy, as shown in multiple recently published studies.^18–20^ The median length of hospital stay decreased with the introduction of enhanced recovery programs and was 9 days for patients after an uncomplicated esophagectomy in the Netherlands, probably contributing to the decreased maximum hospital days to achieve textbook outcome.^21^^,^^22^ Although these changes to the original textbook outcome seem to follow recent findings regarding esophageal cancer treatment in literature, no actual rationale for the inclusion of each of the items was provided by the responding surgeons.

Some limitations have to be addressed. First, due to the design of this survey no initial consensus was reached for some of the items belonging to one subcategory, and responding experts were contacted once more to make a final vote for inclusion of these items. A different survey set-up would have provided the opportunity for a direct answer on inclusion of these items, although this would have prevented the consulted experts to rely their decision on the results of the first round. Furthermore, not all surgeons responding to the initial survey provided their vote in the second round (88.9%), and although a preferred item from every subcategory could be selected, agreement rates were limited, varying from 38% to 66%. Second, as consulted expert esophageal cancer surgeons were selected from the TIGER study group, a prospective cohort study focusing on the distribution of lymph node metastases in esophageal cancer surgery, a different conception of the optimal lymph node yield might exist compared to gastro-esophageal cancer surgeons in general. However, a survey conducted prior to the initiation of the TIGER study inquiring participating surgeons on the extent of their lymphadenectomy suggests otherwise, as it revealed great variation in the extent of lymphadenectomy between the participating surgeons.^23^ Lastly, another limitation of the current study was the inclusion of predominantly Western surgeons due to the relatively higher rate of non-responders from Asian counties. We hypothesize that the response rate of surgeons originating from the Netherlands was particularly high (90%) due to their familiarity with textbook outcome and its value in current literature.

In conclusion, the current study provided an update of the textbook outcome for esophageal cancer surgery based on international consensus. Quality measures may be subject to change in light of modifications to treatment protocols and accompanying changes in treatment outcomes. This updated textbook outcome should be implemented in quality assurance programs for centers performing esophageal cancer surgery, and could standardize quality measures used internationally. Universal quality measures allow international inter-center comparison, which could function as an extra stimulus for further surgical quality improvement.

## Notes

Authors’ contributions

Study conception and design: Marianne C. Kalff, Mark I. van Berge Henegouwen, and Suzanne S. Gisbertz; Data acquisition and analysis: Marianne C. Kalff; Interpretation of data: Marianne C. Kalff, Mark I. van Berge Henegouwen and Suzanne S Gisbertz; Drafting the manuscript: Marianne C. Kalff: Revising the manuscript: Marianne C. Kalff, Mark I. van Berge Henegouwen and Suzanne S. Gisbertz; All authors approved the final version.

## References

[1] Schmidt H M, Gisbertz S S, Moons J et al. Defining benchmarks for transthoracic Esophagectomy: a multicenter analysis of total minimally invasive Esophagectomy in low risk patients. Ann Surg 2017; 266(5): 814–21. 28796646 10.1097/SLA.0000000000002445

[2] Low D E, Kuppusamy M K, Alderson D et al. Benchmarking complications associated with Esophagectomy. Ann Surg 2019; 269(2): 291–8. 29206677 10.1097/SLA.0000000000002611

[3] Glatz T, Höppner J. Is there a rationale for structural quality assurance in esophageal surgery? Visc Med 2017; 33(2): 135–9. 28560229 10.1159/000458454 PMC5447175

[4] Busweiler L A D, Schouwenburg M G, van Berge Henegouwen M I et al. Textbook outcome as a composite measure in oesophagogastric cancer surgery. Br J Surg 2017; 104(6): 742–50. 28240357 10.1002/bjs.10486

[5] Courrech Staal E F W, Wouters M W J M, Boot H, Tollenaar R A E M, van Sandick J W. Quality-of-care indicators for oesophageal cancer surgery: a review. Eur J Surg Oncol 2010; 36(11): 1035–43. 20846818 10.1016/j.ejso.2010.08.131

[6] Van Der Werf L R, Wijnhoven B P L, Fransen L F C et al. A National Cohort Study evaluating the association between short-term outcomes and long-term survival after esophageal and gastric cancer surgery. Ann Surg 2019; 270(5): 868–76. 31634182 10.1097/SLA.0000000000003520

[7] Van Workum F, Stenstra M H B C, Berkelmans G H K et al. Learning curve and associated morbidity of minimally invasive Esophagectomy: a retrospective multicenter study. Ann Surg 2019; 269(1): 88–94. 28857809 10.1097/SLA.0000000000002469

[8] Kalff M C, Vesseur I, Eshuis W J et al. The association of textbook outcome and long-term survival after esophagectomy for esophageal cancer. Ann Thorac Surg 2020. doi: 10.1016/j.athoracsur.2020.09.035. 33221197

[9] Levy J, Gupta V, Amirazodi E et al. Gastrectomy case volume and textbook outcome: an analysis of the population registry of esophageal and stomach tumours of Ontario (PRESTO). Gastric Cancer 2020; 23(3): 391–402. 31686260 10.1007/s10120-019-01015-w

[10] Priego P, Cuadrado M, Ballestero A, Galindo J, Lobo E. Comparison of laparoscopic versus open gastrectomy for treatment of gastric cancer: analysis of a textbook outcome. J Laparoendosc Adv Surg Tech 2019; 29(4): 458–64. 10.1089/lap.2018.0489 30256171

[11] Clavien P A, Barkun J, de Oliveira M L et al. The Clavien-Dindo classification of surgical complications: five-year experience. Ann Surg 2009; 250(2): 187–96. 19638912 10.1097/SLA.0b013e3181b13ca2

[12] Samson P, Puri V, Broderick S, Patterson G A, Meyers B, Crabtree T. Adhering to quality measures in esophagectomy is associated with improved survival in all stages of esophageal cancer. Ann Thorac Surg 2017; 103(4): 1101–8. 28109569 10.1016/j.athoracsur.2016.09.032 PMC5444909

[13] Chang A C, Kosinski A S, Raymond D P et al. The Society of Thoracic Surgeons composite score for evaluating esophagectomy for esophageal cancer. Ann Thorac Surg 2017; 103(5): 1661–7. 28385375 10.1016/j.athoracsur.2016.10.027

[14] Hagens E, Van Berge Henegouwen M I, Gisbertz S. Distribution of lymph node metastases in esophageal carcinoma [TIGER study]: study protocol of a multinational observational study. BMC Cancer 2019; 19(1): 662. 31272485 10.1186/s12885-019-5761-7 PMC6610993

[15] Low D E, Alderson D, Cecconello I et al. International consensus on standardization of data collection for complications associated with esophagectomy: esophagectomy complications consensus group (ECCG). Ann Surg 2015; 262(2): 286–94. 25607756 10.1097/SLA.0000000000001098

[16] Donabedian A . Evaluating the quality of medical care. Milbank Q 2005; 83(4): 691–729. 16279964 10.1111/j.1468-0009.2005.00397.x PMC2690293

[17] Fransen L F C, Berkelmans G H K, Asti E et al. The effect of postoperative complications after minimally invasive esophagectomy on long-term survival: an international multicenter cohort study. Ann Surg 2020; doi: 10.1097/SLA.0000000000003772. 31972650

[18] Chen D, Mao Y, Xue Y, Sang Y, Liu D, Chen Y. Does the lymph node yield affect survival in patients with esophageal cancer receiving neoadjuvant therapy plus esophagectomy? A systematic review and updated meta-analysis. EClinicalMedicine 2020; 25: 100431. 32775970 10.1016/j.eclinm.2020.100431 PMC7397690

[19] Sihag S, Nobel T, Hsu M et al. A more extensive lymphadenectomy enhances survival following neoadjuvant chemoradiotherapy in locally advanced esophageal adenocarcinoma. Ann Surg 2020; Publish Ahead of Print. 10.1097/SLA.0000000000004479 PMC8114152 33201124

[20] Visser E, Markar S R, Ruurda J P, Hanna G B, van Hillegersberg R. Prognostic value of lymph node yield on overall survival in esophageal cancer patients: a systematic review and meta-analysis. Ann Surg 2019; 269(2): 261–8. 29794846 10.1097/SLA.0000000000002824

[21] Voeten D M, van der Werf L R, van Sandick J W, van Hillegersberg R, van Berge Henegouwen M I. Length of hospital stay after uncomplicated esophagectomy. Hospital variation shows room for nationwide improvement. doi: 10.1007/s00464-020-08103-4. PMC8523439 33104919

[22] Markar S R, Karthikesalingam A, Low D E. Enhanced recovery pathways lead to an improvement in postoperative outcomes following esophagectomy: systematic review and pooled analysis. Dis Esophagus 2015; 28(5): 468–75. 24697876 10.1111/dote.12214

[23] van Rijswijk A S, Hagens E R C, van der Peet D L, van Berge Henegouwen M I, Gisbertz S S. Differences in esophageal cancer surgery in terms of surgical approach and extent of lymphadenectomy: findings of an international survey. Ann Surg Oncol 2019; 26(7): 2063–72. 30903323 10.1245/s10434-019-07316-9 PMC6545175

